# Attitudes of family medicine physicians toward the Turkish-validated 12-item attitude scale for the clinical nutrition care process: a national cross-sectional analysis

**DOI:** 10.1590/1806-9282.20260175

**Published:** 2026-07-10

**Authors:** Mustafa Köksal, Neslişah Gürel Köksal, Ilker Sengul

**Affiliations:** 1 Giresun Provincial Directorate of Health – Giresun, Turkey.; 2 Giresun University Training and Research Hospital, Department of Family Medicine – Giresun, Turkey.; 3 Giresun University, Faculty of Medicine, Thyroidology Unit – Giresun, Turkey.; 4 Giresun University, Faculty of Medicine, Division of Endocrine Surgery – Giresun, Turkey.; 5 Giresun University, Faculty of Medicine, Department of General Surgery – Giresun, Turkey.

**Keywords:** Nutrition, Malnutrition, Nutritional assessment, Primary health care, Gastrointestinal

## Abstract

**OBJECTIVE:**

Malnutrition in older adults drives functional decline, higher morbidity, and rising healthcare costs. Although family medicine specialists are well placed to manage community nutritional risk, their clinical engagement is often limited. The aim of this study was to evaluate their attitudes toward the clinical nutrition care process and identify professional factors influencing these views.

**METHODS:**

We conducted a national cross-sectional survey among family medicine specialists in Turkey using the Turkish-validated 12-item Attitude Scale for the Clinical Nutrition Care Process. The scale evaluates two primary dimensions: Physician duties (Factor 1) and Non-physician duties (Factor 2), with total scores ranging from 12 to 60. Professional characteristics, including work setting, clinical nutrition training, and self-perceived competence, were also assessed. Statistical associations were explored using Welch’s t-test, Welch analysis of variance, and Pearson correlation.

**RESULTS:**

Overall, 410 specialists participated (65.6% female; mean age 37.0±5.9 years). Although 71.2% (n=292) had clinical nutrition training, only 30.0% (n=123) used nutrition screening tools, and 32.4% (n=133) reported self-perceived competence in malnutrition management. The mean total attitude score was 40.76±6.48, moderately positive. Work setting affected attitudes (p=0.025): home health scored higher than outpatient clinics (42.23±6.09 vs. 40.04±6.88). Screening-tool use and self-perceived competence were significantly associated with more favorable attitudes toward interprofessional duties (Factor 2) (p=0.008; p=0.016).

**CONCLUSION:**

Despite generally positive attitudes, a major gap remains between clinical perception and delivering nutritional care in primary practice. Low use of screening tools and limited perceived competence indicate an urgent need for interventions that combine hands-on training with institutional support to improve community nutritional management.

## INTRODUCTION

Malnutrition in the aging population is a critical clinical and public health challenge, directly contributing to functional deterioration, increased susceptibility to disease, and elevated mortality rates^1^. Beyond its clinical ramifications, the economic burden on health and social services is substantial, yet malnutrition remains frequently under-recognized and inadequately addressed in primary care settings^1^. Evidence indicates that early identification of nutritional risk, followed by the administration of oral nutritional supplements (ONS), can significantly improve clinical outcomes and reduce complications^2^.

Family medicine specialists occupy a central role in the healthcare system, serving as the first point of contact for community-dwelling older adults and patients with chronic conditions. They are uniquely positioned to detect early signs of nutritional vulnerability and coordinate comprehensive care. However, persistent gaps in knowledge and attitudes regarding malnutrition have been documented in primary care^3^. Barriers to routine nutritional screening include systemic constraints such as time pressure, competing clinical priorities, and variable access to specialized training^4,5^. Furthermore, qualitative research suggests that uncertainty regarding professional roles and clinical responsibilities often hinders effective nutritional intervention^4^.

While educational initiatives are essential for enhancing clinical competence, their efficacy is maximized when they are context-sensitive and supported by a facilitating clinical environment^6^. Structured nutritional curricula have been shown to improve self-efficacy among clinicians^7^, and updated primary care interventions can significantly bolster knowledge^8^. To optimize these initiatives, it is vital to utilize validated instruments to assess clinicians’ perceived roles and attitudes. The 12-item Attitude Scale for the Clinical Nutrition Care Process provides a robust framework for evaluating these perspectives, specifically distinguishing between physician-centric and interprofessional duties^9^. This national study sought to evaluate the attitudes of family medicine specialists in Turkey toward the clinical nutrition care process and to delineate the sociodemographic and professional factors that shape these attitudes.

## METHODS

### Study design and participant selection

This national, descriptive cross-sectional study was conducted among family medicine specialists actively practicing in Turkey. All procedures were conducted in accordance with the Declaration of Helsinki, and informed consent was obtained from all participants. Data was collected via an electronic survey platform (Google Forms), distributed through established professional networks and digital communication channels. A snowball sampling strategy was employed to ensure broad geographic and institutional representation. Based on an estimated population of 4,100 family medicine specialists, the minimum sample size was determined to be 352 (95%CI, 5% margin of error) to ensure statistical power.

### Instrumentation and data collection

Eligible participants were board-certified family medicine specialists in active practice who provided informed consent. Exclusion criteria included non-physician personnel, students, retired physicians, or those in purely administrative roles. The primary instrument was the Turkish-validated 12-item Attitude Scale for the Clinical Nutrition Care Process of Hospitalized Patients for Physicians^9^. This 5-point Likert scale (1=strongly disagree to 5=strongly agree) comprises two factors: Factor 1 (Physician duties; eight items) and Factor 2 (Non-physician duties; four items). Total scores range from 12 to 60, with higher scores reflecting more favorable attitudes. Additionally, a comprehensive questionnaire captured sociodemographic and professional metrics, including work setting, years in practice, prior nutrition-specific training, use of screening tools, and self-assessed competence in malnutrition management.

### Statistical analysis

Continuous variables are presented as mean±standard deviation, median (interquartile range), and range, while categorical data are reported as frequencies and percentages. Internal consistency of the scale was evaluated using Cronbach’s alpha. Comparative analyses utilized Welch’s t-test for binary groups and Welch analysis of variance for multi-group comparisons, supplemented by adjusted post-hoc testing where appropriate. The correlation between age/experience and attitude scores was assessed using Pearson’s correlation coefficient. All statistical tests were two-tailed, with significance set at p<0.05.

## RESULTS

### Cohort characteristics and clinical practices

A total of 410 family medicine specialists participated (65.6% female; mean age 37.0±5.9 years; mean years in practice 10.7±5.3). Most respondents were affiliated with tertiary-care institutions (55.6%) and outpatient clinics (52.2%). Notably, while 71.2% (n=292) had received prior clinical nutrition training—primarily through residency (42.7%) and courses/seminars (48.5%)—only 30.0% (n=123) reported the routine use of nutritional screening tools, and a mere 32.4% (n=133) perceived themselves as competent in managing malnutrition (Table 1).

**Table 1:** Sociodemographic characteristics of family medicine specialists (n=410).

Characteristic	Category	n (%)/Mean±SD	Median (IQR)	Min–max
Age, years		37.00±5.92	37 (32–41)	26–50
Years in practice		10.66±5.26	11 (6–15)	1–23
Sex	Female	269 (65.6)		
	Male	141 (34.4)		
Hospital level	Primary care (1st level)	87 (21.2)		
	Secondary (2nd level)	95 (23.2)		
	Tertiary (3rd level)	228 (55.6)		
Work setting	Outpatient clinic	214 (52.2)		
	Inpatient ward	108 (26.3)		
	Home health	88 (21.5)		
Any clinical nutrition training	Yes	292 (71.2)		
	No	118 (28.8)		
Training source*	Medical school	75 (18.3)		
	Residency	175 (42.7)		
	Course/Seminar	199 (48.5)		
Uses a nutrition screening tool	Yes	123 (30.0)		
	No	287 (70.0)		
Self-perceived competence in malnutrition	Yes	133 (32.4)		
	No	277 (67.6)		

Values are presented as mean±SD, median (IQR), or n (%).

* Training source categories are not mutually exclusive. SD: standard deviation; IQR: interquartile range.

### Attitude scale outcomes

The mean total score on the Clinical Nutrition Care Attitude Scale was 40.76±6.48 (observed range: 21–52). Subscale analysis revealed mean scores of 27.26±4.77 for Physician Duties (Factor 1) and 13.50±2.25 for Non-physician Duties (Factor 2). The instrument demonstrated high internal consistency (Cronbach’s α=0.851 for the total scale; α=0.817 for Factor 1; α=0.611 for Factor 2) (Table 2).

**Table 2:** Mean total scores of the Clinical Nutrition Care Attitude Scale and its subdimensions among family medicine specialists (n=410).

Scale	Items (n)	Possible range	Observed range	Mean±SD	Median (IQR)	Cronbach’s α
Total scale	12	12–60	21–52	40.76±6.48	42 (36–45)	0.851
Factor 1 (Physician duties)	8	8–40	15–35	27.26±4.77	29 (25–31)	0.817
Factor 2 (Non-physician duties)	4	4–20	6–18	13.50±2.25	14 (12–16)	0.611

Items 1, 6, 7, 8, and 12 are negatively worded and were reverse-coded before score calculation. Factor 1 comprises items 1, 2, 3, 4, 5, 9, 10, and 11; Factor 2 comprises items 6, 7, 8, and 12. SD: standard deviation; IQR: interquartile range.

### Determinants of clinician attitudes

Total attitude scores did not differ significantly by sex, hospital level, or prior training (all p>0.05). However, the clinical work setting emerged as a significant determinant of attitude scores (p=0.025 for total; p=0.030 for Factor 1; p=0.028 for Factor 2). Specialists working in home health services exhibited significantly more positive attitudes (Total: 42.23±6.09) than those in outpatient clinics (Total: 40.04±6.88) (Table 3). Furthermore, the routine use of screening tools (p=0.008) and higher self-perceived competence (p=0.016) were significantly associated with more favorable attitudes toward interprofessional duties (Factor 2), although these factors did not translate into significantly higher total scores. No significant correlations were observed between age or professional longevity and attitude scores (Table 3).

**Table 3:** The effect of sociodemographic characteristics on the Clinical Nutrition Care Attitude Scale and its subdimensions (n=410).

Variable	Category	n	Total score	Factor 1 score	Factor 2 score	p (total)	p (F1)	p (F2)
Sex	Female	269	40.53±6.84	27.06±5.03	13.47±2.36	0.288	0.199	0.742
	Male	141	41.21±5.71	27.66±4.21	13.55±2.04			
Hospital level	Primary care (1st level)	87	39.92±7.44	26.52±5.55	13.40±2.38	0.422	0.223	0.770
	Secondary (2nd level)	95	41.22±5.91	27.81±4.33	13.41±2.28			
	Tertiary (3rd level)	228	40.89±6.30	27.32±4.61	13.57±2.20			
Work setting	Outpatient clinic	214	40.04±6.88	26.70±5.13	13.34±2.32	0.025	0.030	0.028
	Inpatient ward	108	40.99±5.76	27.64±4.24	13.35±2.11			
	Home health	88	42.23±6.09	28.17±4.29	14.06±2.18			
Any clinical nutrition training	Yes	292	41.12±6.42	27.52±4.64	13.59±2.25	0.084	0.093	0.180
	No	118	39.88±6.56	26.62±5.03	13.26±2.24			
Uses a nutrition screening tool	Yes	123	41.52±6.68	27.58±5.00	13.94±2.17	0.128	0.397	0.008
	No	287	40.44±6.37	27.13±4.67	13.31±2.26			
Self-perceived competence in malnutrition	Yes	133	41.35±6.73	27.47±5.01	13.88±2.19	0.210	0.547	0.016
	No	277	40.48±6.34	27.16±4.65	13.31±2.26			
Age (years)		410	0.006 (p=0.900)	0.008 (p=0.866)	0.000 (p=0.997)			
Years in practice		410	0.014 (p=0.779)	0.017 (p=0.733)	0.004 (p=0.933)			

p-values for categorical variables were obtained using Welch’s t-test (2 groups) or Welch ANOVA (≥3 groups). Age and years in practice were assessed using Pearson correlation. Two-sided p-values are reported.

## DISCUSSION

Our national analysis reveals that family medicine specialists in Turkey maintain a moderately positive disposition toward the clinical nutrition care process. However, a profound disconnect exists between these attitudes and clinical implementation: despite widespread training exposure (71.2%), the utilization of objective screening tools remains low (30.0%), and self-perceived competence is limited (32.4%). Given the established link between malnutrition and adverse outcomes in older adults, these findings identify a critical opportunity for systemic improvement in primary care^1^. The efficacy of early nutritional intervention, particularly through ONS, further emphasizes the need for a more robust screening infrastructure^2^.

These results align with international observations suggesting that while primary care physicians acknowledge the importance of nutrition, they often lack the confidence or systemic support to implement it effectively^3^. The barriers identified—including time constraints and role ambiguity—reflect a global challenge in integrating nutrition into the workflow of general practice^4,5^. Interestingly, our finding that prior training did not correlate with higher total attitude scores suggests that current educational models may be too theoretical or disconnected from the practical realities of primary care. For education to be transformative, it must be integrated into clinical workflows and supported by an environment that facilitates nutritional screening and intervention^6,7,8^.

The use of a validated scale originally designed for inpatient settings provided a rigorous framework for our assessment, although the lower reliability of the “Non-physician duties” subscale in our cohort may reflect the unique interprofessional dynamics of primary care versus hospital settings^9^. The higher attitude scores observed among home health physicians are particularly noteworthy. These clinicians frequently manage frail, homebound patients where the clinical impact of malnutrition is highly visible, likely fostering a greater sense of clinical ownership and perceived relevance^10,11^. This suggests that exposure to high-risk populations may be a more potent driver of positive attitude than traditional didactic training.

Furthermore, the association between screening tool use and positive attitudes toward interprofessional collaboration (Factor 2) suggests that clinicians who engage in objective assessment are more likely to value a multidisciplinary approach to care. This is consistent with qualitative evidence indicating that malnutrition management is heavily influenced by how clinicians perceive their roles within the broader healthcare team^12,13^. Successful implementation in primary care requires not only individual competence but also clear referral pathways and institutional support^14^.

Our study benefits from a large, national sample and the use of a validated instrument, yet it is not without limitations. The cross-sectional design and reliance on self-reported data may introduce bias, and the snowball sampling method might limit the generalizability of the findings. Future research should focus on longitudinal interventions that combine practical training with workflow optimization and interprofessional support.

## CONCLUSION

Family medicine specialists in Turkey demonstrate a receptive attitude toward nutritional care, yet this has not translated into routine clinical practice. The low rates of objective screening and limited perceived competence signify a critical gap in primary care delivery. To bridge this divide, it is imperative to move beyond simple didactic education toward the implementation of integrated, multidisciplinary nutritional care pathways that are seamlessly embedded within the primary care environment.

## Data Availability

The datasets generated and/or analyzed during the current study are available from the corresponding author upon reasonable request.
